# Neutrophil extracellular trap from Kawasaki disease alter the biologic responses of PBMC

**DOI:** 10.1042/BSR20200928

**Published:** 2020-09-07

**Authors:** Yang Jing, Meng Ding, Jiuyuan Fu, Yanping Xiao, Xianghua Chen, Qingyun Zhang

**Affiliations:** 1 Department of Vascular Surgery, Affiliated Hospital of Chengde Medical University, No. 36, Nanyingzi Street, Shuangqiao District, Chengde, Hebei 067000, China; 2 Laboratory Medicine, Affiliated Hospital of Chengde Medical University, No. 36, Nanyingzi Street, Shuangqiao District, Chengde, Hebei 067000, China; 3 Obstetrics Department, Affiliated Hospital of Chengde Medical University, No. 36, Nanyingzi Street, Shuangqiao District, Chengde, Hebei 067000, China

**Keywords:** HIF-1α, Kawasaki disease, neutrophil extracellular trap, PBMCs, VEGF-A

## Abstract

Kawasaki disease (KD), also known as mucocutaneous lymph node syndrome, is an acute systemic vasculitis syndrome that mainly occurs in infants under 5 years of age. In the current manuscript, we were aiming to analyze the role of neutrophil extracellular traps (NETs) in the pathogenesis of KD, especially their interplay with peripheral blood mononuclear cells (PBMCs). Neutrophils were exposed to 20 nM phorbol myristate acetate (PMA), we found that neutrophils of KD patients were more likely to form NETs compared with healthy controls (HCs). Furthermore, PBMCs were cultured with NETs for 24 h, and we observed that NETs significantly increased the cell viability, suppressed cell apoptosis, and enhanced the pro-inflammatory cytokines production and NF-κB activation in PBMCs from KD patients. In addition, with the stimulation of NETs, the expression of vascular endothelial growth factor A (VEGF-A) and hypoxia-inducible factor-1α (HIF-1α) were increased, which were related with the pathological mechanism of KD. At last, we examined the activation of phosphoinositide 3 kinase (PI3K)/Akt signaling, and we found NETs treatment obviously enhanced the activation of PI3K and Akt. In conclusion, these findings suggested that the formation of NETs may alter the biologic responses of PBMC and affect the vascular injury in KD.

## Introduction

Kawasaki disease (KD) is a serious multisystem vasculitis, it mainly occurs in kids under 5 years, especially those of Asian and Pacific Island descent [1]. Coronary artery abnormalities and myocardial ischemia can be observed in KD patients [2]. Furthermore, some children suffer from myocardial infarction. KD has replaced rheumatic fever as the main cause of childhood acquired heart disease [3]. Although the KD mortality and coronary complications have been significantly declined by extensive application of the combination of intravenous gammaglobulin (IVIG) injection and aspirin [4]. But the etiology and mechanism are not clear yet. It is presumed at present that the features of KD are consistent with infectious disease and autoimmune disease [5].

Neutrophils are the most important inflammatory cells in the early inflammatory response of innate immunity. Recently, it has been reported that in addition to phagocytosis, neutrophils could lose the integrity of their inner membrane and release network of chromatin fibers, which can immobilize and kill invading microbes. These structures were named neutrophil extracellular traps (NETs) [6]. NETs release self-DNA decorated with antimicrobial proteins, such as MPO, PR3, cathelicidin, LL37 etc [7]. Recently, abnormal NETs generation was reported to be correlated with autoimmune diseases and played cytotoxic effect on vascular endothelial cells [8]. Persistent endothelial injury always leads to endothelial hyperplasia, vascular endothelial growth factor A (VEGF-A) was reported to play essential roles in angiogenesis as well as chemotactic function of immune cells [9], and these details provide us the insight that VEGF-A could be an indicator of inflammation-related diseases. Indeed, it has been reported that in the serum of KD patients, VEGF-A level was concerned with the development of coronary arterial lesions (CALs) [10].

Because KD is associated with vascular injury and autoimmune response, and NETs can also affect the development of autoimmune diseases and vascular injury, therefore, in the present manuscript, we investigate the effect of synthesis of NETs in the pathogenesis of KD, and due to the critical role of peripheral blood mononuclear cells (PBMCs) in KD and blood immune responses, we mainly detected the effect of NETs on biologic response of PBMCs. We found that neutrophils of KD patients were more likely to form NETs compared with healthy control group. Furthermore, we observed that NETs treatment increased the cell viability, the pro-inflammatory cytokines production and NF-κB activation in PBMCs from KD patients. Moreover, after NETs stimulation, the activation of phosphoinositide 3 kinase (PI3K) and Akt was markedly enhanced. These findings indicated that NETs could alter the biologic responses of PBMC and suggested NETs as potential target for the KD treatment.

## Materials and methods

### Patients

The study included 12 KD patients (all in acute period) and 6 healthy control (HC) patients from May 2017 to August 2018 in Affiliated Hospital of Chengde Medical University. Related facilities are shown in Table 1. KD diagnosis were performed according to a previous report [11]. Informed consents were obtained from all patients, the research protocol was approved by the Ethics Committee of the Affiliated Hospital of Chengde Medical University.

**Table 1:** Data of KD patients and HCs

Characteristics	KD, *n*=12	HCs, *n*=6
	*n*=12	*n*=6
Male/female	8/4	4/2
Age at onset (m)	14–46 (21)	12–41 (23)
CAL (+/−)	0/19	0/6
CRP (mg/dl)	2.8–21.8 (6.8)	ND
White blood cell count (× 10^9^/l)	9.7–26.3 (14.88)	6.03–11.33 (9.02)
Neutrophils (%)	39–88 (69)	33–62 (42)
Platelet count (× 10^9^ /l)	215–837 (365)	244–410 (328)
Na (mmol/l)	133–148 (140)	ND
AST (IU/l)	19–254 (33)	28–55 (42)
Duration of fever (d)	5–11 (7)	ND

*n*, number of patients. Data from each group show that there were no significant differences between the KD patients and the control groups. Numbers indicates range (medium) in each variable. Abbreviations: AST, aspartate transaminase; CRP, C-reactive protein. ND, no data.

### Cell culture

According to the manufacturer’s instructions, PBMCs were obtained from patients’ whole blood by Ficoll-Paque (Sigma, St. Louis, MO) gradient centrifugation. PBMCs were cultured in RPMI 1640 containing 10% fetal bovine serum (FBS) and incubated at 37°C in a 5% CO_2_ condition.

### NETs formation

NETs formation was as follows: neutrophils were obtained from whole blood by centrifugation through poly-morphprep gradient centrifugation according to the manufacturer’s instructions (Thermo Scientific). Neutrophils were cultured at 37°C for 30 min, following 20 nM phorbol myristate acetate (PMA; Sigma–Aldrich) treatments for another 2 h. NETs formation was observed under a fluorescent microscope and was quantified as previously reported [7].

### PBMCs culture with NETs

Neutrophils were treated with 20 nM PMA and incubated at 37°C. Two hours later, the medium with straw was removed carefully and added with fresh medium. After incubating at 37°C for 30 min, the wash step was repeated and the bottom NETs were collected. After centrifugation (1200 rpm for 5 min), the supernatant was applied to PBMCs cultured with 10% FBS in RPMI 1640. For the Mock group, PBMCs were added with RPMI 1640 with 10% FBS. At 24 h after the addition of conditioned NETs media, the PBMCs were collected for the following assays.

### Cell viability and LDH examination

CCK8 assay was performed to detect cell viability (Beyotime Technology, Shanghai, Beijing). LDH level was examined by LDH Cytotoxicity Assay Kit (Thermo Scientific, Waltham, MA).

### RNA analysis

Total RNA was extracted with TRIzol reagent. GAPDH was chosen as internal control, and qPCR experiments were performed by using SYBR Green Master Mix (Applied Biosystems, Foster City, CA, U.S.A.) and evaluated by the 2^−ΔΔC_T_^ method [12]. Sequences of primers used in the RT-PCR studies are shown in Table 2.

**Table 2:** Primer sequences

Genes	Forward (5′–3′)	Reverse (5′–3′)
TNF-α	CTA AGA GGG AGA GAA GCA	AGA GGC TGA GGA ACA AGC
IL-6	CCT TCG GTC CAG TTG CCT TCT	CCA GTG CCT CTT TGC TGC TTT
IL-8	TTG CCA AGG AGT GCT AAA GAA	GCC CTC TTC AAA AAC TTC TCC
HIF-1α	GCG CGA ACG ACA AGA AAA AGA	GTG GCA ACT GAT GAG CAA GC
VEGF-A	CTT GCC TTG CTG CTC TAC CT	GCA GTA GCT GCG CTG ATA GA
GAPDH	AAT GAC CCC TTC ATT GAC	TCC ACG ACG TAC TCA GCG

### Western blot

Western blot was examined as reported [9]. The antibodies for IκBα (#4814), phospho-IκBα (#2859), IKKβ (#8943), phospho-IKKβ (#2078), p65 (#8242), phospho-p65 (#3033), phospho-PI3K (#4228), PI3K (#4249), phospho-Akt (#9611), Akt (#4691), hypoxia-inducible factor-1α (HIF-1α; #36169), cleaved-caspase3 (#9664), caspase3 (#9662) and GAPDH (#5174) were all bought from Cell Signaling Technology (Cell Signaling Technology Inc, Beverly, U.S.A.). The antibodies for Bcl-2 (#sc509) and Bax (#sc20067) were obtained from Santa Cruz Biotechnology (Santa Cruz Biotechnology, Santa Cruz, U.S.A.).

### Enzyme-linked immunosorbent assay

VEGF-A enzyme-linked immunosorbent assay (ELISA) kits were used to evaluate VEGF-A concentrations (Immuno-Biologic Laboratories, Fujioka, Japan) according to the manufacturer’s instructions. Interleukin (IL)-8, IL-6, tumor necrosis factor-α (TNF-α) ELISA kits (R&D Systems, Minneapolis, U.S.A.) were also used in the present study.

### Statistical analysis

Data are presented as mean ± SD. Statistical significance was determined by using ANOVA test. A probability of less than 0.05 was considered to be statistically significant.

## Results

### Neutrophils of KD patients were more likely to form NETs

**Figure 1:**
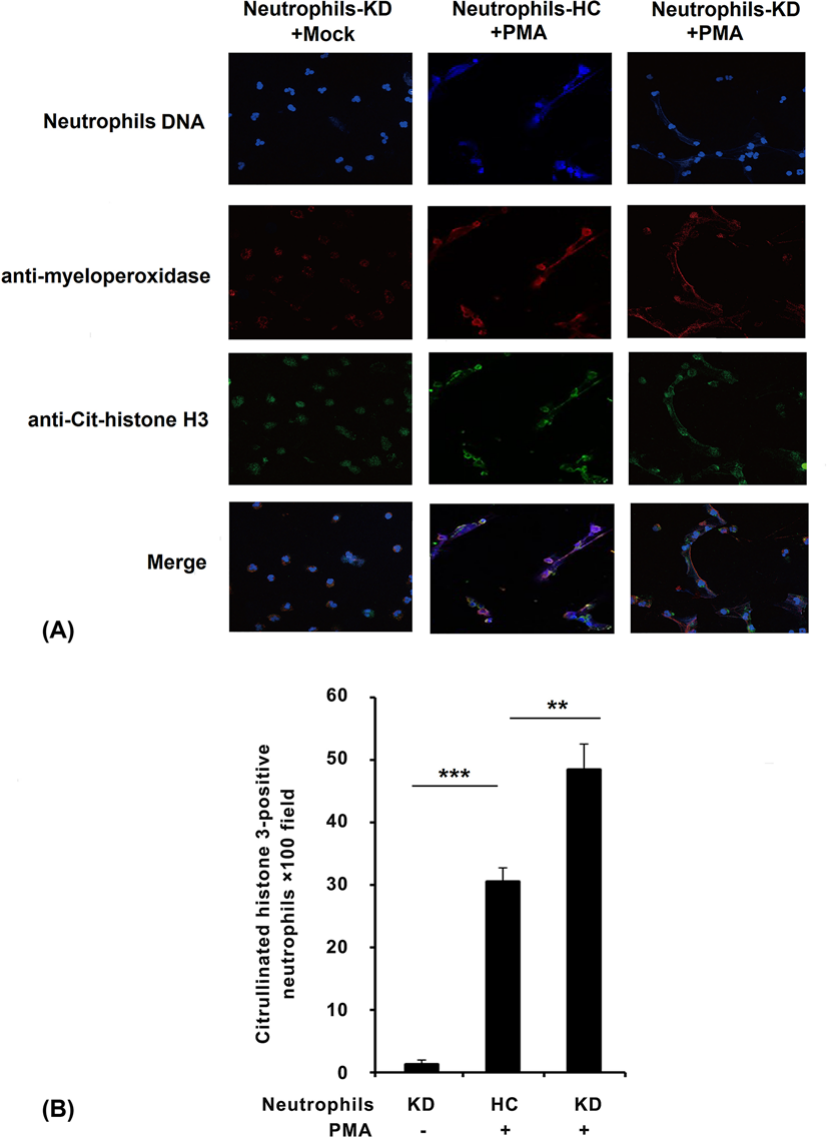
Neutrophils of KD patients were more likely to form NETs (**A**) Neutrophils from patients were incubated for 30 min, followed by treatment with 20 nM PMA and incubated for another 2 h, then processed for immunofluorescence under a fluorescent microscope. (**B**) NETs formation was quantified by counting the citrullinated histone 3-positive cells per ×100 power field of view. Data from five random fields of view (×100) were subjected to quantitative analysis. The data are representative of three independent experiments. **P<0.01, ***P<0.001.

Human peripheral blood neutrophils were isolated from KD patients or HC patients. Neutrophils of all groups were exposed to 20 nM PMA to perform extended NETs, and NETs formation was examined by fluorescent microscope. The images show merged neutrophil DNA (blue), histone H3 (green), and myeloperoxidase (red) staining (Figure 1A). The findings corresponded with some researches and suggested that PMA induced the formation of NETs (Figure 1A). Furthermore, compared with neutrophils of HC patients after PMA stimulation, we found that neutrophils of KD patients were more likely to form NETs when stimulated with PMA, which can be observed in the results (Figure 1B).

### Effect of NETs on cell viability and apoptosis in PBMCs

**Figure 2:**
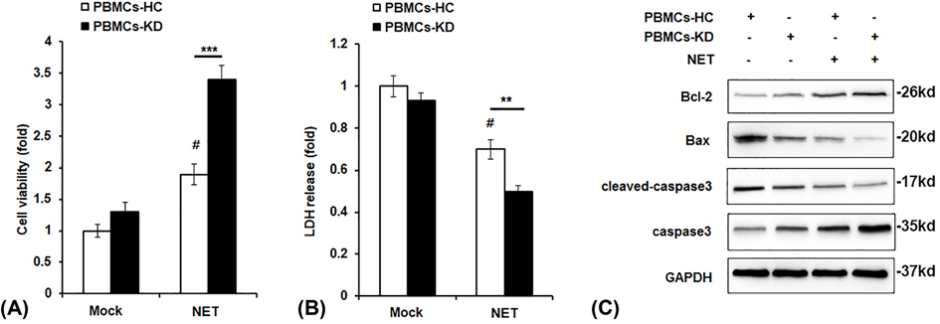
Effect of NETs on cell viability and apoptosis in PBMCs PBMCs of HC or KD patients were incubated with NETs from KD patients for 24 h. Cell viability (**A**), LDH release (**B**), and protein levels of Bax, Bcl-2 and cleaved-caspase3 (**C**) were examined by CCK8 assay (**A**), LDH cytotoxicity assay (**B**), and Western blot assay (**C**). **P<0.01 versus PBMCs-HC+NETs group, ***P<0.001 versus PBMCs-HC+NETs group, ^#^*P*<0.05 versus PBMCs-HC+control group.

To investigate to effect of NETs on PMBCs of KD, we incubated the PBMCs of HC or KD patients with NETs from KD patients for 24 h. First, we examined the effect of NETs on PBMCs cell viability. As shown in Figure 2A, NETs treatment significantly enhanced cell viability in both PBMCs-HC and PBMCs-KD groups; most importantly, PBMCs-KD group showed higher cell viability compared with PMBC-HC after NETs stimulation. Lactate dehydrogenase (LDH) release is a widely used indicator of cell damage, and it was found to be markedly decreased in PBMCs-KD group with NETs incubation (Figure 2B). In addition, we detected the expression of apoptosis-related proteins, and we found that cleaved-caspase3 and Bax expression were decreased while Bcl-2 expression was increased in NETs-treated PBMC-KD group (Figure 2C).

### Effect of NETs on cytokines production and NF-κB activation in PBMCs

**Figure 3:**
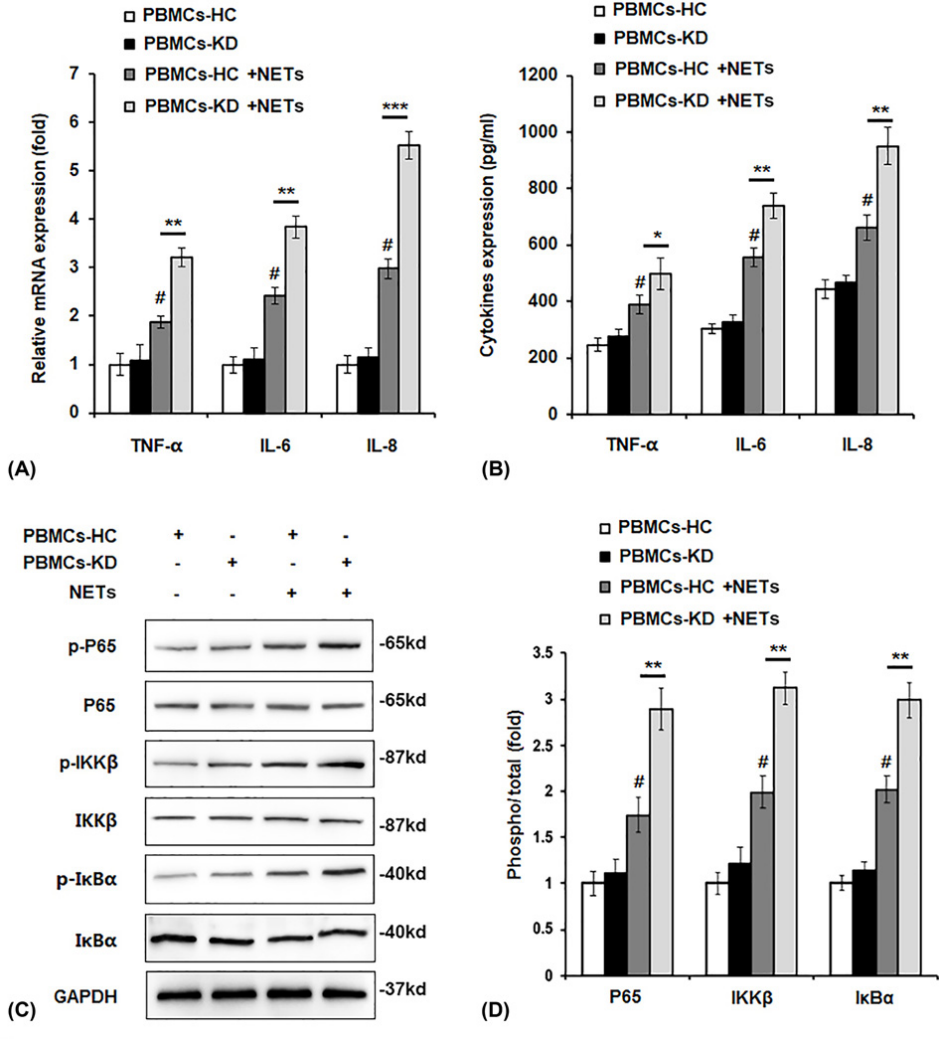
Effect of NETs on cytokines production and NF-κB activation in PMBCs PBMCs of HC or KD patients were incubated with NETs from KD patients for 24 h. mRNA level (**A**), protein level (**B**) of pro-inflammatory cytokines expression, protein levels of p-P65, p-IKKβ, p-IκBα (**C**) were detected by qPCR assay (**A**), ELISA (**B**), and Western blot assay (**C**). (**D**) Quantification of protein level of p-p65, p-IKKβ, p-IκBα in (C) by using the ImagePro-Plus software. *P<0.05, **P<0.01 versus PBMCs-HC+NETs group, ***P<0.001 versus PBMCs-HC+NETs group, ^#^*P*<0.05 versus PBMCs-HC+control group.

It is reported that up to 80% of KD patients develop a subclinical myocarditis within the first few weeks of disease [13], therefore pro-inflammatory cytokines production was detected. As shown in Figure 3A,B, pro-inflammatory cytokines such as TNF-α, IL-6, and IL-8 were all up-regulated by NETs treatment. We hypothesized if NETs affected pro-inflammatory cytokines expression through NF-κB pathway, and we found that phosphorylation of p65, IKKβ, and IκBα was obviously increased by NETs stimulation, in addition, we found the phosphorylated levels were higher in PBMCs-KD group compared with PBMCs-HC group (Figure 3C,D).

### Effect of NETs on expression of VEGF-A and HIF-1α in PBMCs

**Figure 4:**
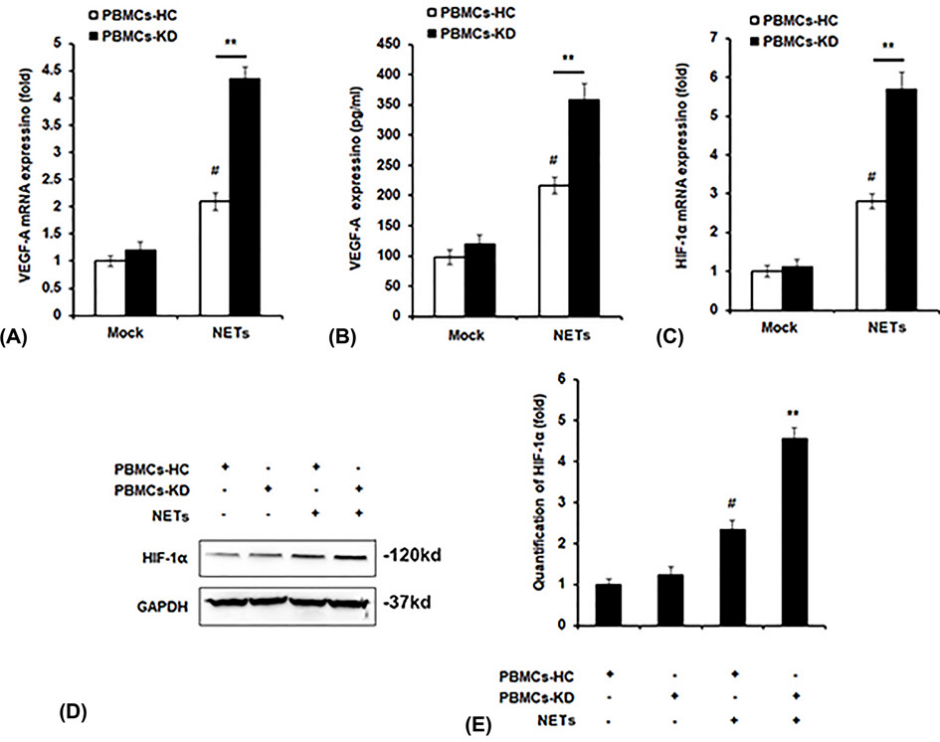
Effect of NETs on expression of VEGF-A and HIF-1α in PMBCs PBMCs of HC or KD patients were incubated with NETs from KD patients for 24 h. mRNA level of VEGF-A (**A**), protein level of VEGF-A (**B**), mRNA level of HIF-1α (**C**) and protein level of HIF-1α (**D**) were detected by qPCR assay (**A,C**), ELISA (**B**) and Western blot assay (**D**). (**E**) Quantification of HIF-1α in (**D**) by using the Image-Pro Plus software. **P<0.01 versus PBMCs-HC+NETs group, ^#^*P*<0.05 versus PBMCs-HC+control group.

VEGF-A is a key molecule in the pathogenesis of KD. Therefore, mRNA and protein levels of VEGF-A in PBMCs were examined, and we found that the levels of VEGF-A in PBMCs-KD and NETs co-cultured group were obviously elevated than other groups (Figure 4A,B). HIF-1α has been reported to be essential in regulating the type and magnitude of inflammatory and innate immune responses produced by neutrophils and macrophages, which also activates the transcription of VEGF [14], thus we examined the expression of HIF-1α after NETs treatment. We found both the mRNA and protein expression of HIF-1α was increased by NETs, and the levels of HIF-1α were markedly increased in PBMCs-KD group compared with PBMC-HC group after NETs stimulation (Figure 4C–E).

### Effect of NETs on PI3K/Akt activation in PBMCs

**Figure 5:**
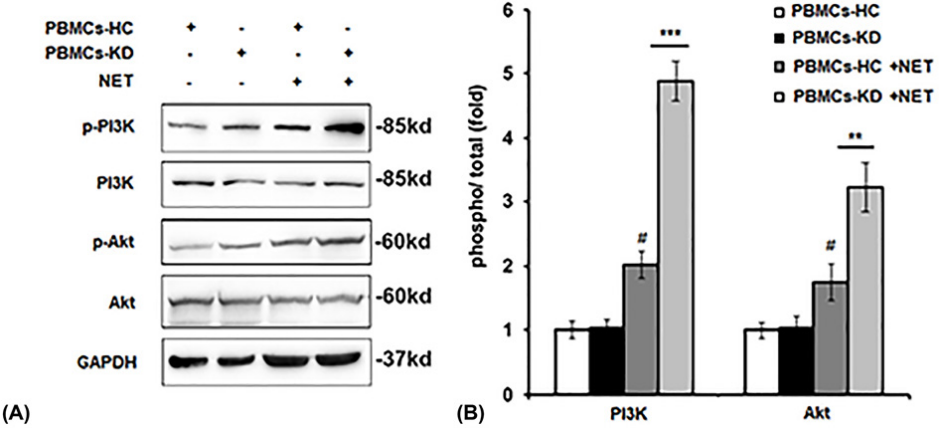
Effect of NETs on PI3K/Akt activation in PMBCs PBMCs of HC or KD patients were incubated with NETs from KD patients for 24 h. Protein levels of p-PI3K and p-Akt were detected by Western blot assay (**A**). Quantification of protein level of p-PI3K and p-Akt in (**A**) by using the Image-Pro Plus software (**B**). **P<0.01 versus PBMCs-HC+NETs group, ***P<0.001 versus PBMCs-HC+NETs group, ^#^*P*<0.05 versus PBMCs-HC+control group.

To explore the mechanism that how NETs regulate the level of HIF-1α, we investigate the effect of NETs on PI3K/Akt activation, which is the main signaling pathway to regulate HIF-1α expression. As shown in Figure 5A,B, we found NETs treatment increased the phosphorylation of PI3K and Akt, especially in PBMCs-KD group.

## Discussion

KD is the most common acute systemic vasculitis syndrome complicated by the development of coronary artery abnormalities [15]. Immune activation is the significant feature of acute phase of KD [16]. At present, it is speculated that the specific cause of KD is closely related to the dysregulation of innate immunity, but the accurate mechanisms still remain largely unknown [17].

After pathogen infection, it was found that neutrophils released a large amount of histone and DNA and formed a kind of network structure with the function of immobilizing and killing pathogenic microorganisms [18]. Previous researches named the network structure as NETs, and it was exactly a new method of killing pathogenic microorganisms and first proposed in 2004. Recently, NETs have been widely studied as a major component of innate immunity [19].

NETs have unique position in the development of disease. In most cases, the production of NETs is beneficial to the innate immunity, but recent studies have also found that NETs promotion could have double-sided effects [20]. Previous studies have shown that NETs have a direct cytotoxic effect on vascular endothelial cells, and abnormal regulation of NETs is related to the production of pathogenic auto-antibodies, leading to the autoimmune diseases development [21]. However, the effects of NETs on pathogenesis of KD, especially their interplay with PBMCs remain largely unknown.

PMA could induce the formation of NETs [22]. In the current study, we found that neutrophils of KD patients were more likely to form NETs compared with HC when stimulated with PMA, which can be observed in the results (Figure 1). We found that after stimulation by NETs, cell viability was significantly up-regulated, and cell apoptosis was attenuated (Figure 2). We also observed that pro-inflammatory cytokines production was increased by NETs treatment, together with the activation of NF-κB signaling (Figure 3). Previous study showed that VEGF-A plays a crucial role in the development of KD development [16]. It mainly functions by influencing angiogenesis and chemotaxis of immune cells [23]. Recently, Saito et al. showed that serum levels of VEGF-A in KD patients are related with the development of CALs [24]. Furthermore, we detected VEGF-A levels. The results suggested that after NETs treatment, VEGF-A secretion by PBMCs from KD patients was higher (Figure 4A–C). HIF-1α has been reported as the transcription factor of VEGF-A [14], and it has been reported that HIF-1α plays essential roles in the formation of mast cell extracellular traps [25]. In the current study, we found that NETs markedly induced HIF-1α expression in NETs-treated PBMCs, especially in PBMCs-KD groups (Figure 4D,E), which suggested that HIF-1α could play great roles in the regulation of NETs induced pro-inflammatory cytokines production and VEGF-1 expression. At last, we found that NETs increased the phosphorylation of PI3K and Akt (Figure 5), leading to the activation of PI3K/Akt signaling pathway, therefore increased the expression of HIF-1α, and activated the following physiological processes.

These results suggest that neutrophils in KD patients are more likely to be stimulated to form NETs than those in HCs. In addition, NETs could significantly promote the activation of PI3K/Akt and NF-κB signaling in PBMCs of KD patients, resulting in the up-regulation of HIF-1α and VEGF expression, and the occurrence of more serious inflammatory reactions, as well as the corresponding changes in cell survival and apoptosis properties. This provides us with a new insight into KD treatment. By interfering with the formation of NETs and weakening the storage of NETs, maybe we could effectively alleviate the degree of inflammation in KD patients, and then alleviate the damage of vascular endothelial cells induced by KD.

In conclusion, for the first time, we illustrated the effect of NETs on PBMCs of KD which might give us a hint of novel therapeutic agents for KD.

## Abbreviations

CAL: coronary arterial lesion
CCK8: Cell Counting Kit-8
ELISA: enzyme-linked immunosorbent assay
FBS: fetal bovine serum
GAPDH: glyceraldehyde-3-phosphate dehydrogenase
HC: healthy control
HIF-1α: hypoxia-inducible factor-1α
IκBα: inhibitor of NF-κB α
IKKβ: inhibitor kappa B kinase β
IL: interleukin
KD: Kawasaki disease
NET: neutrophil extracellular trap
PBMC: peripheral blood mononuclear cell
PI3K: phosphoinositide 3 kinase
PMA: phorbol myristate acetate
qPCR: quantitative Real-time PCR
RT-PCR: real-time PCR
TNF-α: tumor necrosis factor-α
VEGF-A: vascular endothelial growth factor A
